# Postcolonoscopy Followup Recommendations: Comparison with and without Use of Polyp Pathology

**DOI:** 10.1155/2014/683491

**Published:** 2014-08-27

**Authors:** Shiva K. Ratuapli, Suryakanth R. Gurudu, Mary A. Atia, Michael D. Crowell, Sarah B. Umar, M. Edwyn Harrison, Jonathan A. Leighton, Francisco C. Ramirez

**Affiliations:** Division of Gastroenterology and Hepatology, Department of Medicine, Mayo Clinic Arizona, 13400 East Shea Boulevard, Scottsdale, AZ 85259, USA

## Abstract

*Background.* Appropriate recommendations for a followup exam after an index colonoscopy are an important quality indicator. Lack of knowledge of polyp pathology at the time of colonoscopy may be one reason that followup recommendations are not made. *Aim.* To describe and compare the accuracy of followup recommendations made at colonoscopy based on the size and number of polyps with recommendations made at a later date based on actual polyp pathology. *Methods.* All patients who underwent screening and surveillance colonoscopy from March, 2012, to August, 2012, were included. Surveillance recommendations from the endoscopy reports were graded as “accurate” or “not accurate” based on the postpolypectomy surveillance guidelines established by US Multisociety Task Force on Colon Cancer. Polyp pathology was then used to regrade the surveillance recommendations. *Results.* Followup recommendations were accurate in 759/884 (86%) of the study colonoscopies, based upon size and number of polyps with the assumption that all polyps were adenomatous. After incorporating actual polyp pathology, 717/884 (81%) colonoscopies had accurate recommendations. *Conclusion.* In our practice, the knowledge of actual polyp pathology does not change the surveillance recommendations made at the time of colonoscopy in the majority of patients.

## 1. Introduction

Postpolypectomy surveillance constitutes 20% of the colonoscopies [1] performed, thereby contributing to a significant amount of health care expenditure [2, 3]. Prior to the development of postpolypectomy surveillance guidelines, which were based on the National Polyp Study in 1997, annual surveillance was a common practice. Since then, guidelines recommend the surveillance interval be based on risk of polyps found at index colonoscopy [4], rather than intense surveillance irrespective of polyp type.

In 2006, the US Multisociety Task Force on colorectal cancer (USMSTF) issued postpolypectomy surveillance guidelines [4] based on number of polyps, polyp size, and pathology as shown in Table 1. Subsequently in 2012, the USMSTF added surveillance recommendations for serrated lesions to the previous guidelines [5].

However several recent studies [6–8] have reported poor adherence to guidelines among gastroenterologists and primary care physicians. Lack of awareness of established guidelines [9] and intentional noncompliance due to personal preferences, as well as poor quality of bowel preparation [10] have been suggested as possible reasons for poor adherence to guidelines. Lack of polyp pathology at the time of colonoscopy, especially in an open access system, may be another reason that appropriate followup recommendations are not made.

Repeat surveillance colonoscopy earlier than recommended may increase healthcare costs, and delayed surveillance colonoscopy may lead to an interval cancer. Identifying and eliminating the factors that deter adherence to established guidelines are important. Decreasing or eliminating the need to rely on polyp pathology [11] may encourage endoscopists to make more accurate recommendations at the time of the colonoscopy. There is a scarcity of clinical data on this subject. Therefore the aim of this study was to describe and compare the accuracy of the follow-up recommendations made using number, size and educated guess of pathology of polyps at the time of colonoscopy with recommendations based on actual polyp pathology.

## 2. Materials and Methods

The study was approved by the institutional review board of Mayo Clinic, as a quality improvement project. Endoscopy reports and electronic medical records of all the patients who underwent colonoscopies from March, 2012, to August, 2012, were reviewed. Only patients who underwent outpatient screening and surveillance colonoscopy were included in the study. Patients with poor bowel preparation, colonic malignancy, hereditary polyposis syndromes, incomplete exams, and large polyps requiring short term followup were excluded. Data on patient demographics, indication for procedure, quality of bowel preparation, polyp characteristics (number, size, and type of polyp) and anatomical location, and completion of colonoscopy were recorded. Information on whether surveillance recommendations were made at the time of colonoscopy as documented in the procedure report was also recorded.

All colonoscopies were performed either by an attending gastroenterologist or by fellows under direct supervision of the attending gastroenterologist. Complete colonoscopy was defined as intubating the cecum, identifying the appendiceal orifice and ileocecal valve. The quality of bowel preparation was rated by endoscopists based on five prespecified criteria as follows: “excellent,” “good,” “fair-adequate,” “inadequate,” or “poor.”

The first step was to grade the initial recommendations taking into consideration the size and number of polyps and assuming that all removed polyps were adenomatous. Surveillance recommendations made by the endoscopists, based on the number and size of polyps at the time of colonoscopy were graded as “accurate” or “not accurate” based on the 2006 postpolypectomy surveillance guidelines issued by US Multisociety Task Force on colorectal cancer. The definitions of “accurate” and “not accurate” recommendations are listed in Table 2. For purposes of this study, the recommendations that gave a* range of time *but included the guideline recommendation were graded as accurate. The overall accuracy of surveillance recommendations was calculated by adding the accurate recommendations that gave a* range of time, that is, 3–5 years* and the accurate recommendations that gave a* specific time*. Early and late exams were defined as those that were recommended to be performed before and after the guideline recommended time, respectively. Patients with a* range of time* recommendations were categorized into two groups: (a) recommendations with a potential for early colonoscopy [i.e., for a correct recommendation of 5 years, 3–5 years; for a correct recommendation of 10 years: 5–10 years] (b) recommendations with potential for late colonoscopy [i.e., for a correct recommendation of 3 years, 3–5 years; for a correct recommendation of 5 years, 5–10 years] Polyp pathology was then reviewed, and adenomatous polyps were used to regrade the endoscopist's recommendations, using the same definitions for “accurate” and “not accurate” recommendations.

Standard high definition colonoscopes (Olympus America, Center Valley, PA) were used for all the procedures, most of which were performed under moderate sedation with choice of intravenous fentanyl, midazolam, or meperidine as per the preference of the performing endoscopist. Polyethylene glycol based bowel preparations were used in all of the patients.

## 3. Statistical Analysis

Data were entered manually and statistically assessed using IBM SPSS version 20.0 (SPSS Inc., Chicago, IL). Data distributions were evaluated using SPSS Explore and Descriptive functions along with stem-and-leaf plots and histograms. Frequency distributions were evaluated for all categorical variables. Summary statistics included point estimates and standard deviations for all continuous variables and number of patients and percentages for all categorical variables.

## 4. Results

### 4.1. Baseline Characteristics

**Figure 1:**
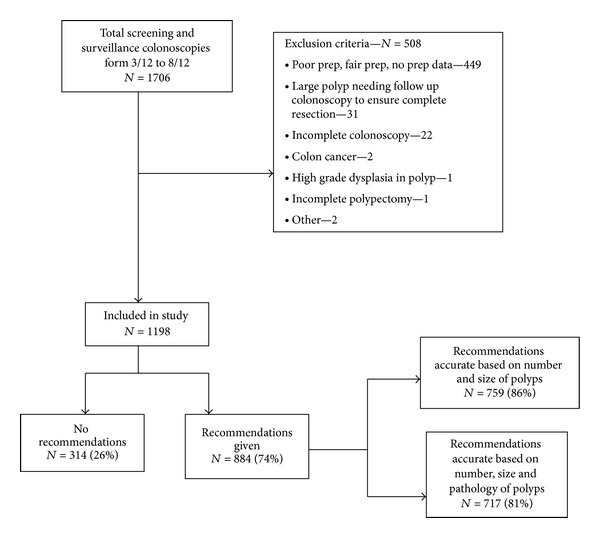
CONSORT diagram.

**Figure 2:**
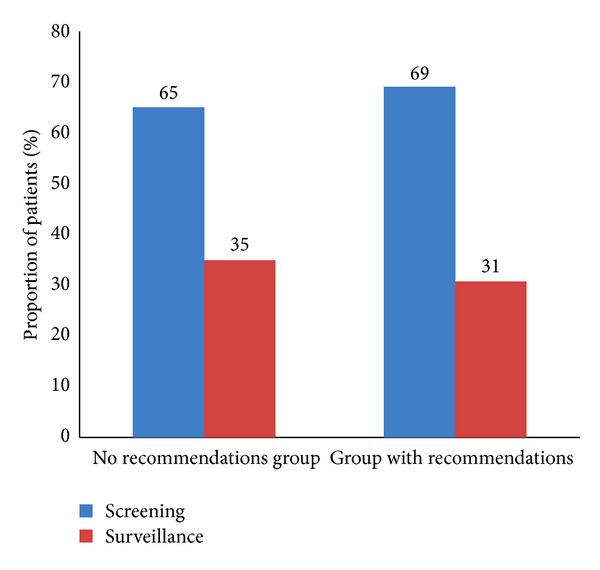
Distribution of screening and surveillance colonoscopies between the “no recommendations group” and “group with recommendations”.

Between March and August of 2012, a total of 1706 screening and surveillance colonoscopies were performed by 21 attending gastroenterologists and 7 gastroenterology fellows. After applying exclusion criteria, 1198 patients were included in the study as shown in Figure 1. Followup colonoscopy recommendations were not made in 26% (314) of the patients at the time of entering the endoscopic report. This group had a higher proportion of surveillance colonoscopies compared to the group where recommendations were given at the time of colonoscopy (Figure 2). The remaining 74% (884) patients were included in the final analysis. The mean age of the patients was 60 ± 9 years, and 48% (422) were women. The overall polyp and adenoma detection rate for this cohort of patients were 62% and 44%, respectively.

### 4.2. Accuracy of Surveillance Recommendations Based on Mode of Assessment

**Figure 3:**
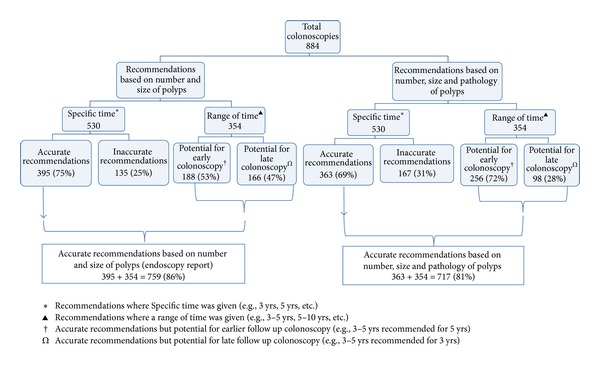
Accuracy of recommendations using two modes of assessment.

Accurate surveillance recommendations were made at the time of colonoscopy, based on number, size (and an educated guess of pathology) in 86% (759/884) of the patients. Based on actual pathology results, accurate recommendations were made in 81% (717/884) of the patients. Of these accurate recommendations, a* range of time* (e.g., 3–5 years for 5 years, 5–10 years for 10 years) was recommended instead of a* specific time*, in half of the patients, but this time range included the time recommended by the guidelines (Figure 3).

**Figure 4:**
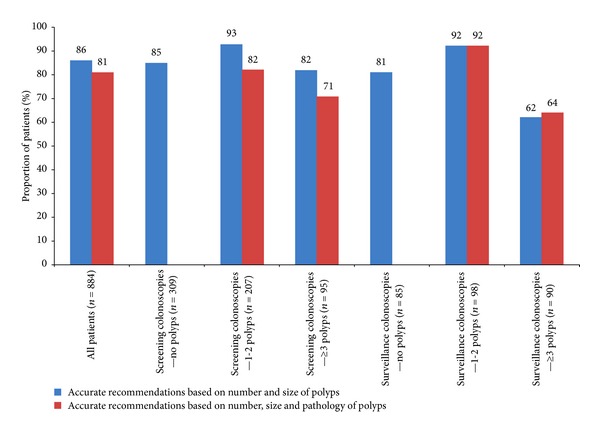
Accuracy of recommendations using two modes of assessment, in various subgroups.

**Figure 5:**
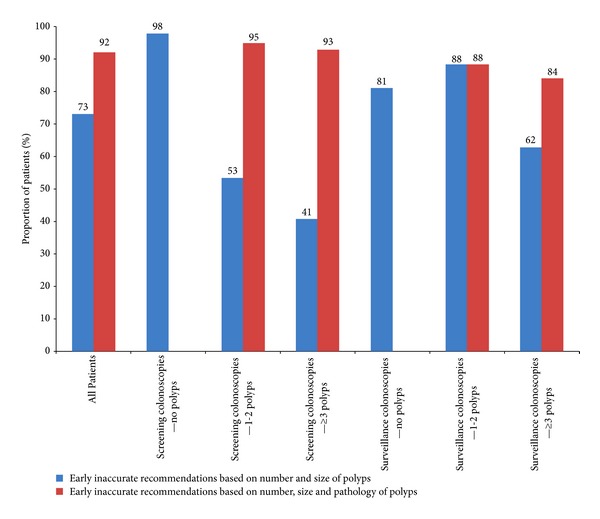
Distribution of inaccurate recommendations for an early colonoscopy using two modes of assessment in various subgroups.

Subgroup analysis of the accuracy of recommendations at the time of the colonoscopy showed that the actual pathology decreased the proportion of accurate recommendations in screening but not surveillance colonoscopies as shown in Figure 4. Analysis of the inaccurate recommendations, using the two modes of assessment showed that the majority of these inaccurate recommendations were for an earlier interval than suggested by guidelines: 73% based on number and size of polyps, 92% based on number, size, and pathology of polyps (Figure 5). When polyp pathology was used to regrade the recommendations, the proportion of inaccurate recommendations was higher Figure 5.

## 5. Discussion

Our study suggests that actual polyp pathology does not change the initial surveillance interval recommendations at the time of colonoscopy in the majority of the patients. Knowing the polyp pathology changed recommendations in only 5% of our study patients. Our study results should increase the confidence and compliance in making appropriate followup surveillance interval recommendations at the time of the colonoscopy, rather than waiting for polyp pathology.

While confirming polyp pathology is important, it is not necessary for recommending the next interval colonoscopy. The hesitancy of making recommendations without pathology likely leads to low adherence among endoscopists in following current guidelines and/or amending the initial report. Similar to our study, three studies [12–14] have assessed accuracy of surveillance recommendations made at the time of colonoscopy. Gupta et al. [13] used the morphological features of polyps found during colonoscopies performed by 6 endoscopists, to predict future surveillance intervals, rather than the actual recommendations made by the endoscopist, and predicted correct surveillance interval in 83.2% of the 410 study patients. Similarly, Ignjatovic et al. [12] and Rex [14] used morphological characters of polyps during colonoscopy to predict surveillance intervals correctly in 98% and 94%, respectively. The accuracy rate of surveillance recommendations made without polyp pathology in our study (86%) was similar to that reported by Gupta et al. and lower than those reported by Ignjatovic et al. and Rex. Our study differs from the reported studies [12–14] in that we compared the original surveillance recommendations made by the performing colonoscopists from the initial endoscopy reports with the surveillance recommendations obtained by histopathological assessment of polyps. Additionally, our study included a larger number of study patients and a higher number of performing endoscopists, compared to the other studies.

Similar accuracy of surveillance recommendations with and without using polyp pathology, as seen in our study supports American Society of Gastrointestinal Endoscopy's PIVI (Preservation and Incorporation of Valuable Endoscopic Innovations) [11] to promote endoscopic technologies that aid in optical diagnosis of diminutive colon polyp histology. Our results could encourage endoscopists to either resect and discard or leave in place small diminutive polyps [13, 15], if high definition endoscopes with narrow band imaging were used in optical diagnosis of small polyps [16, 17].

The common cause for making inaccurate recommendations in our study was recommending an early colonoscopy than suggested by the guidelines. When polyp pathology was used to grade the accuracy of recommendations, 92% of the inaccurate recommendations were made for an earlier colonoscopy. An earlier than recommended followup colonoscopy exam can be considered detrimental for the healthcare system since it implies an unplanned and most likely unnecessary expense whereas a recommendation for late followup colonoscopy can be considered potentially detrimental for the patient since it would delay the diagnosis of significant colonic lesions arising from polyps.

There are other reasons that surveillance interval recommendations made after colonoscopy may not be accurate. As reported in other studies [9, 18–22], lack of awareness of guidelines or unwillingness of endoscopists to adhere to guidelines may be some of the reasons for not making accurate recommendations. Other possible reasons include the status of the bowel preparation at the time of colonoscopy, the endoscopist's level of comfort with current interval guidelines, unavailability of the patient's family history, and the lack of knowledge of the endoscopist's own adenoma detection rate in different segments of the colon. Additionally, we speculate that lack of confidence of the visual diagnosis of polyp pathology at the time of making recommendations may have led our endoscopists to recommend a colonoscopy early than necessary.

The limitation of this study is that it was retrospective in nature. Classifying recommendations that gave a* range of time* as accurate may not be ideal, as this may give the endoscopist or primary care provider enough room for interpretation leading more likely to opt for an earlier interval rather than the longer one. When grading the endoscopist's recommendation, all polyps were considered to be adenomatous, as it is not possible to know which polyps were thought to be hyperplastic or adenomatous by the endoscopist. However, it is reasonable to assume that the endoscopist would only remove polyps that had an appearance suspicious for adenomatous polyps. With this in mind, we assumed retrospectively that all polyps were considered adenomatous. Despite these limitations, our study was strengthened by its large sample size and inclusion of endoscopists with varied levels of experience, thereby increasing the generalizability of our results.

In conclusion, our study suggests that accurate surveillance guidelines can be recommended at the time of colonoscopy without knowing actual polyp pathology. In this cohort of patients, surveillance recommendations made at the time of colonoscopy based on number and size of polyps were accurate in 86% of patients and the surveillance interval after knowledge of polyp pathology did not change the recommendations in majority of patients. Periodic reviews of current guidelines, improvement of bowel preparation quality, and improvements in the endoscopic imaging technology and awareness of individual endoscopist's polyp and adenoma detection rates in various colonic segments may improve the accuracy of interval recommendations based on visual diagnosis at the time of colonoscopy. Future studies should examine the effect of improving adherence to guidelines to see if an adequate accuracy may be achieved.

## Figures and Tables

**Table 1:** 2006 USMSTF surveillance recommendations.

Baseline colonoscopy findings	Recommended surveillance interval (y)
No Polyps	10
Small (<10 mm) hyperplastic polyps	10
1-2 small (<10 mm) tubular adenomas	5–10
3–10 tubular adenomas	3
Any tubular adenoma ≥10 mm	3
>10 adenomas	<3

**Table 2:** Definitions of “accurate” and “not accurate” recommendations.

Number of Polyps	Accurate recommendations-marked “accurate” if following recommendations made	Inaccurate recommendations-marked “not accurate” if following recommendations made
Screening		
No polyp	10 years, 5–10 years	5 years
1-2 polyps <10 mm	3–5 years, 5 years, 5–10 years	3 years
≥3 polyps	3 years, 3–5 years	<3 years
Any polyp >10 mm	3 years, 3–5 years	<3 years

Surveillance		
No polyp	3–5 years, 5 years	3 years
1-2 polyps <10 mm	3–5 years, 5 years, 5–10 years	3 years
≥3 polyps	3 years, 3–5 years	<3 years
